# Land altitude, slope, and coverage as risk factors for Porcine Reproductive and Respiratory Syndrome (PRRS) outbreaks in the United States

**DOI:** 10.1371/journal.pone.0172638

**Published:** 2017-04-17

**Authors:** Andréia Gonçalves Arruda, Carles Vilalta, Andres Perez, Robert Morrison

**Affiliations:** Department of Veterinary Population Medicine, College of Veterinary Medicine, University of Minnesota, St Paul, Minnesota, United States; Kansas State University, UNITED STATES

## Abstract

Porcine reproductive and respiratory syndrome (**PRRS**) is, arguably, the most impactful disease on the North American swine industry. The Swine Health Monitoring Project (**SHMP**) is a national volunteer initiative aimed at monitoring incidence and, ultimately, supporting swine disease control, including PRRS. Data collected through the SHMP currently represents approximately 42% of the sow population of the United States. The objective of the study here was to investigate the association between geographical factors (including land elevation, and land coverage) and PRRS incidence as recorded in the SHMP. Weekly PRRS status data from sites participating in the SHMP from 2009 to 2016 (n = 706) was assessed. Number of PRRS outbreaks, years of participation in the SHMP, and site location were collected from the SHMP database. Environmental features hypothesized to influence PRRS risk included land coverage (cultivated areas, shrubs and trees), land altitude (in meters above sea level) and land slope (in degrees compared to surrounding areas). Other risk factors considered included region, production system to which the site belonged, herd size, and swine density in the area in which the site was located. Land-related variables and pig density were captured in raster format from a number of sources and extracted to points (farm locations). A mixed-effects Poisson regression model was built; and dependence among sites that belonged to a given production system was accounted for using a random effect at the system level. The annual mean and median number of outbreaks per farm was 1.38 (SD: 1.6), and 1 (IQR: 2.0), respectively. The maximum annual number of outbreaks per farm was 9, and approximately 40% of the farms did not report any outbreak. Results from the final multivariable model suggested that increments of swine density and herd size increased the risk for PRRS outbreaks (P < 0.01). Even though altitude (meters above sea level) was not significant in the final model, farms located in terrains with a slope of 9% or higher had lower rates of PRRS outbreaks compared to farms located in terrains with slopes lower than 2% (P < 0.01). Finally, being located in an area of shrubs/ herbaceous cover and trees lowered the incidence rate of PRRS outbreaks compared to being located in cultivated/ managed areas (P < 0.05). In conclusion, highly inclined terrains were associated with fewer PRRS outbreaks in US sow farms, as was the presence of shrubs and trees when compared to cultivated/ managed areas. Influence of terrain characteristics on spread of airborne diseases, such as PRRS, may help to predicting disease risk, and effective planning of measures intended to mitigate and prevent risk of infection.

## Introduction

Porcine reproductive and respiratory syndrome (**PRRS**) causes far-reaching financial losses to the North American swine industry [1]. In recent years, there has been considerable advances on understanding disease transmission features, and evaluating the impact of control and eradication strategies [2]. A decrease in farm-level porcine reproductive and respiratory syndrome virus (**PRRSV**) incidence was observed in the U.S. in 2013/2014 when compared to previous years [3]. However, the incidence appears to have stabilized within the last three years [4], which suggests that the disease is far from being controlled in the country.

A detailed description of factors impairing PRRS control and elimination is available elsewhere [5]. Briefly, one of the reasons why PRRS control has been challenging is that the PRRSV may be transmitted among farms through a variety of routes, including airborne transmission [6], movement of infected pigs [7], and fomites (including both transportation and personnel events; [8, 9]). Furthermore, environmental factors known to affect PRRSV survivability and transmission include ultraviolet light, temperature, and relative humidity [10]; however, the impact of natural environmental features such as land elevation and coverage has never been described for PRRS.

One major effort to provide insights on PRRS occurrence in the United States has been the development of the Swine Health Monitoring Project (**SHMP**), coordinated by the University of Minnesota. This national on-going and expanding producers-led, voluntary monitoring project began recording PRRS status for sow sites across different areas of the United States from 2009, and currently enrolls 42% of the country’s sow population, with 29 participating production systems that share status for one or more diseases (including PRRS, porcine epidemic diarrhea [**PED**] and seneca valley virus). Project participants are required to share farm location and basic demographic information at enrollment, and that the farm veterinarian provides weekly disease status updates. The SHMP database is currently being used as a near real-time disease monitoring tool and as a provider of data for retrospective studies [3, 11, 5].

The objective of this study was to investigate the association between land elevation and coverage, and the incidence of PRRS outbreaks in swine farms across the United States, using data routinely collected at the SHMP. Results will help to understand dynamics of PRRSV transmission in the U.S., ultimately helping to predict risk, and design effective control strategies, for, arguably, one of the most important diseases endemically affecting swine production globally.

## Materials and methods

### Source population and output definition

Sow sites voluntarily participating in the SHMP (n = 706) were assessed in the study here. Data were captured for all herds between 2009 and 2016 (status were provided retrospectively from 2009–2011 and prospectively weekly thereafter). The inclusion criterion was that they shared PRRS status (reporting of the occurrence of new PRRS cases). New PRRS cases were reported weekly by the veterinarian or herd manager via e-mail exchange with the SHMP coordinator. The definition of a new case depended on the veterinarian’s judgement. This decision is influenced by presence of clinical signs in sows and piglets and diagnostic results, often including PRRSV sequence similarity with viruses from previous outbreaks (if any).

### Data collection for variables of interest

Factors hypothesized to influence PRRS risk included swine density in the area, herd size, production system, region, land elevation, and land coverage. Number of pigs, production system, and region were captured from the SHMP database and were provided by the veterinarian and herd manager during site enrollment. A production system was defined as two or more swine sites with a common owner or management structure. Land-related variables and pig density were captured in raster format from a variety of sources.

**Fig 1:**
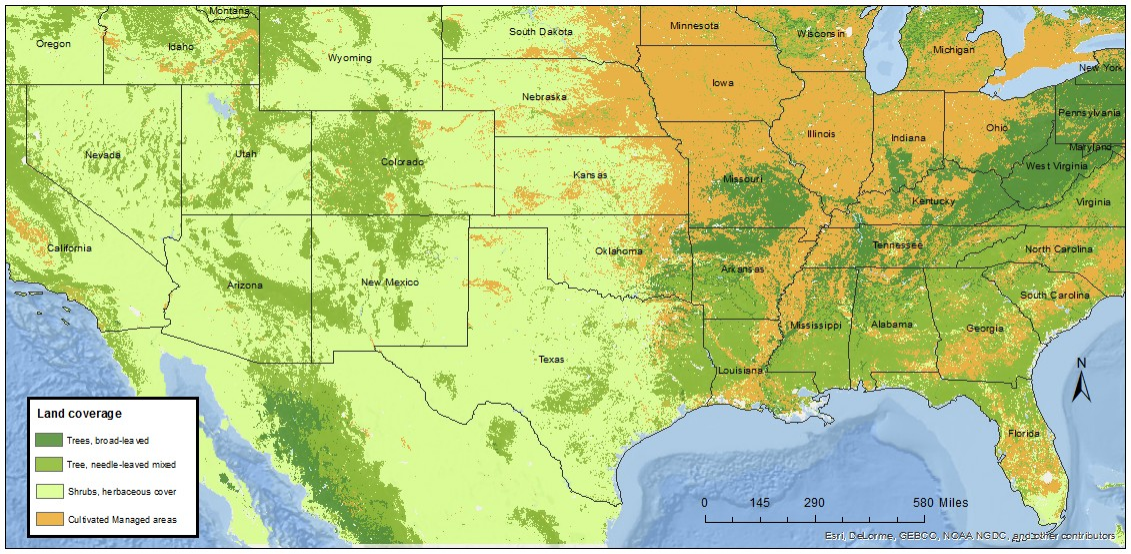
Land coverage raster created using ArcMap 10.2.2. The land coverage raster was obtained from the Global Land Cover 2000 Project (GLC 2000, [12]), coordinated and implemented by the Joint Research Centre (European Commission).

The land coverage raster was obtained from the Global Land Cover 2000 Project (GLC 2000, [12]), coordinated and implemented by the Joint Research Centre (European Commission). The land coverage raster was downloaded with a resolution of 30 arc-seconds, which corresponds to approximately 90 meters at the equator. The original raster was comprised of 22 categories that were grouped accordingly into four broader categories to ensure adequate sample size (Fig 1).

The geographical distribution of the land coverage categories is shown in Fig 1.

**Fig 2:**
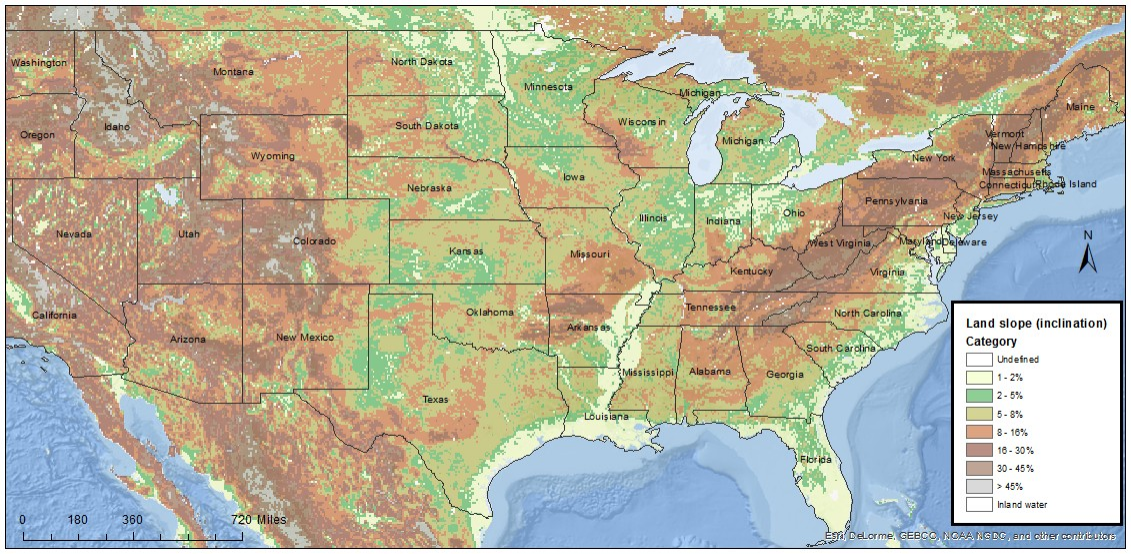
Land slope raster created using ArcMap 10.2.2. Data used for the creation of this map was obtained from the “Derived soil properties” of the FAO-NESCO Soil Map of the world, which aggregates the GTOPO30 dataset with a spatial resolution of 5*5 arc minutes (approximately 10km grids). Information on data processing and equations is available under the “Global Terrain Slope and Aspect Data” reference on the FAO website provided under the reference list [15].

Two rasters were obtained to explore land elevation, namely, altitude and land slope. The altitude raster, expressed in meters above sea level, was obtained from the Shuttle Radar Topography Mission (**SRTM** 30, [13]) dataset in combination with the US Geological Survey (**USGS**) GTOPO30 [14]. The SRTM 30 is comprised of data gathered from the shuttle flown by NASA in February 2000. The resolution level is 30-arc seconds. The land slope raster (Fig 2) was obtained from the “Derived soil properties” of the FAO-NESCO Soil Map of the world, which aggregates the GTOPO30 dataset with a spatial resolution of 5*5 arc minutes (approximately 10km grids). Information on data processing and equations is available under the “Global Terrain Slope and Aspect Data” reference on the FAO website provided under the reference list [15]. A representation of this raster is shown in Fig 2.

**Fig 3:**
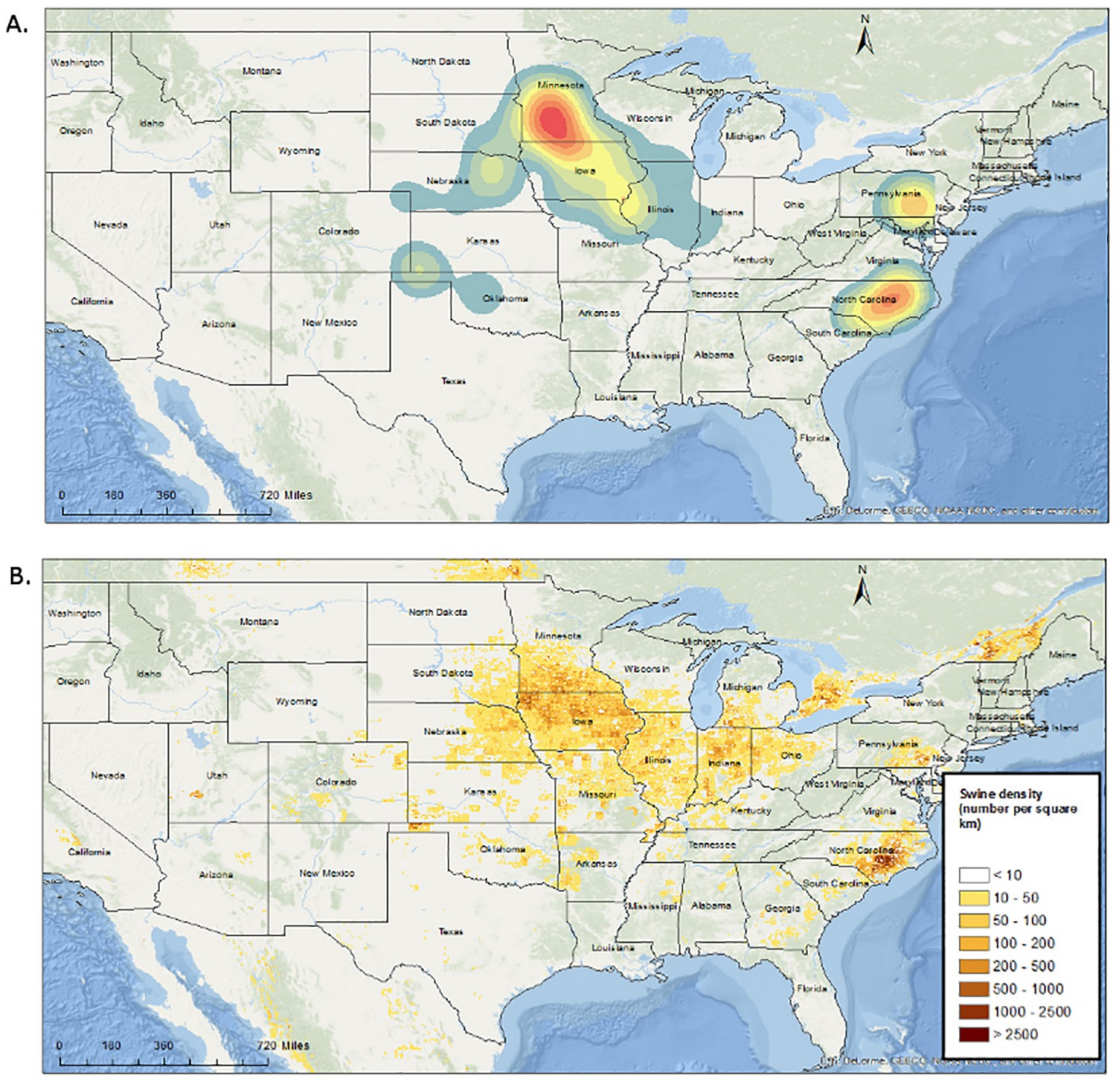
Kernel smoothing of sow sites participating in this study (A); and pig density from FAO projection (2005; B), created using ArcMap 10.2.2. Data for (B) was obtained from the FAO’s GeoNetwork data repository (global livestock densities, modelled data). This raster was predicted for 2005, and adjusted to match FAOSTAT 2005 national totals.

The pig density raster was obtained from the FAO’s GeoNetwork data repository (global livestock densities, modelled data). This raster was predicted for 2005, and adjusted to match FAOSTAT 2005 national totals (Fig 3B). All of these variables were extracted to points (farm locations) using ArcMap 10.2.2.

The main outcome of interest in this study was the number of new PRRS cases (i.e. outbreaks) experienced by sow sites enrolled in the SHMP. This outcome was captured using the number of PRRS outbreaks reported in the dataset (2009–2016). Due to the fact that sow farms participated different amount of time in the SHMP, this number of years was also captured and used as an offset in the model [16].

### Statistical analysis

A mixed-effects Poisson regression model was built using STATA/IC 14.1 (College Station, TX: StataCorp LP). The Poisson model was selected because it is appropriate for modelling counts of relatively rare events (number of outbreaks), and years participating in the project can be easily accounted for by using an offset to adjust for different amounts of time at risk for the study subjects (farms) [16]. Model building steps included first checking for linearity between the continuous variables of interest and the predicted rate. In cases for which the linearity assumption was not met, variables were categorized in the median or quartiles (Table 1).

**Table 1:** Description of exploratory variables of interest and results from univariable analyzes. The main outcome of interest was the counts of PRRS outbreaks at the farm level, and an offset was used to account for different periods participating in data collection (2009–2016).

Variable			Univariable Analysis^1^	
	Category	N^2^ (%)	IRR (SE)^3^	P-value
Pig density^4^	Low	352 (49.9)	Ref	
	High	354 (51.1)	1.97 (0.15)	<0.01
N pigs^5^	Low	343 (48.6)	Ref	
	High	363 (51.4)	1.29 (0.10)	< 0.01
Land altitude^6^	< 185 m	178 (25.2)	0.87 (0.15)	0.43
	186 – 317m	180 (25.5)	1.24 (0.21)	0.19
	318 – 391m	172 (24.4)	0.76 (0.13)	0.11
	> 392m	176 (24.9)	Ref	
Land coverage	Cultivated, managed	473 (67.0)	Ref	
	Shrubs, herbaceous cover	98 (13.9)	0.62 (0.08)	<0.01
	Trees, needle-leaved	62 (8.8)	0.41 (0.08)	<0.01
	Trees, broad-leaved	73 (10.3)	0.03 (0.07)	<0.01
Land slope^7^	<2%	55 (7.8)	Ref	
	2–4%	247 (35.0)	1.11 (0.16)	0.48
	5–8%	301 (42.6)	0.73 (0.11)	0.04
	9–16%	69 (9.8)	0.35 (0.08)	<0.01
	17–30%	34 (4.8)	0.09 (0.05)	<0.01
Region	Illinois	112 (15.9)	Ref	
	Minnesota/ Iowa	228 (32.3)	2.30 (0.31)	<0.01
	North Carolina	118 (16.7)	0.98 (0.36)	0.96
	Nebraska	67 (9.5)	0.67 (0.12)	0.02
	Other	59 (8.4)	0.38 (0.08)	<0.01
	Oklahoma	50 (7.1)	0.92 (0.21)	0.71
	Pennsylvania	72 (10.2)	0.12 (0.04)	<0.01

^1^Generalized mixed Poisson models accounted for clustering of swine sites within production systems using a random effect

^2^Number of swine sites within each category

^3^Incidence rate ratio (standard error)

^4^Categorized in the median (46 pigs/km^2^)

^5^Categorized in the median (2500 pigs/site)

^6^Land altitude measured in meters above sea level, categorized in quartiles

^7^Land inclination, measured in % or degrees

Correlation was checked using the Spearman correlation coefficient, and using a cut-off value of 0.80. Univariable mixed models were built and a conservative P-value of 0.2 was used for screening variables that moved into the full model.

**Fig 4:**
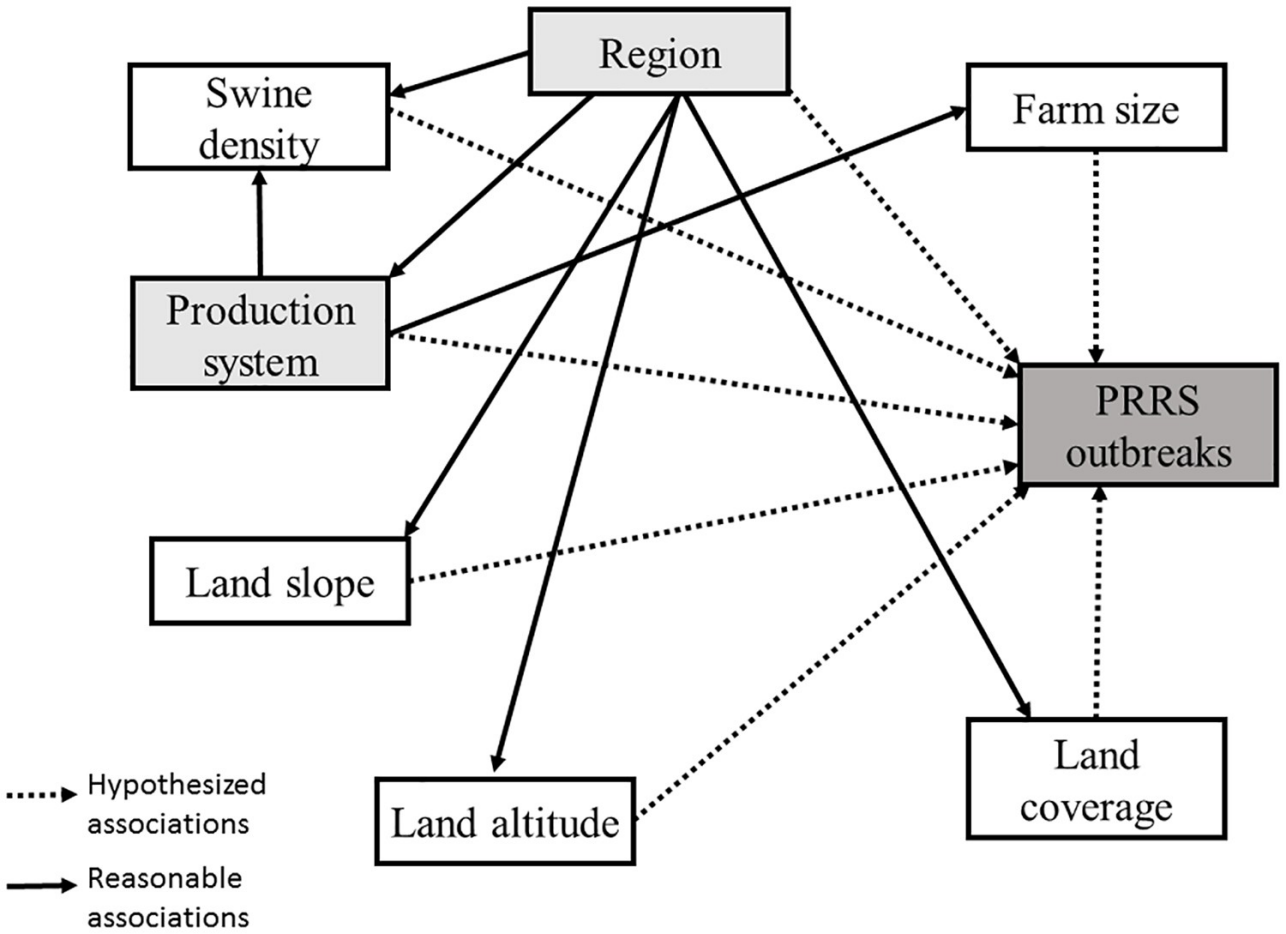
Causal diagram showing the hypothesized and plausible associations between the outcome of interest and investigated exploratory variables.

Finally, the full model was built using a backwards stepwise approach and final statistical significance declared at P < 0.05. Confounders were checked a priori using a causal diagram (Fig 4) and after each variable’s removal. Variables were detected and kept in the model if their removal changed the coefficient of retained variables by 20% or more. Interactions were considered between variables that remained in the final model. Because the clustering of swine sites within production systems was previously shown to be important in the swine industry [9], we tested this hypothesis by fitting and comparing the mixed-effects Poisson model (using production system as a random effect) to a Poisson model that was unadjusted for production system. We evaluated the importance of the random effect parameter by comparing the Akaike Information Criterion (**AIC**) and the Bayesian Information Criterion (**BIC**) from the two models; and by monitoring the change in the regression coefficients of the variables that fitted the final model. The measures of association were obtained as incidence rate ratio (IRR) and statistically significant associations were declared at P < 0.05.

Outliers were identified by checking for large values of Pearson and Ascombe residuals. Best linear unbiased predictors (BLUPs) were estimated and checked for normality [16]. Furthermore, a negative binomial model was also fit and the two nested models were compared using the AIC and the BIC and the model with lower values for those was selected (Poisson model).

Because data was expected to be spatially correlated, the Moran’s I test was used on 1) the response variable; 2) the residuals from a GLM Poisson model accounting for pig density only, and 3) the residuals from the full GLM model. Spatial autocorrelation was declared under the Moran’s I test when P < 0.05. If the spatial correlation detected in the response (model 1) was not found in the model in which pig density only was used as explanatory variable (model 2), then it was assumed that pig density accounted for the spatial dependence of disease risk, and thus, associations detected in the final model (model 3) were assumed to be independent from disease risk spatial clustering [17].

## Results

There were a total of 706 herds included in this study, representing approximately 1,959,918 sows (approximately 33% of the total number of breeding hogs in the United States as estimated by the USDA in 2016 [18]). The breeding sites were located across 19 states of the United States (Colorado, Oklahoma, Kansas, Nebraska, South Dakota, Minnesota, Iowa, Missouri, Kentucky, Indiana, Illinois, Wisconsin, Pennsylvania, Maryland, Virginia, North Carolina, South Carolina, Georgia and Alabama; Fig 3A).

The swine herds belonged to 21 different systems, the mean number of sites per system was 33.62 (SD: 24.5) and the median was 25 (IQR: 32.0). The largest system contributed 92 sites (11.6% of all sites) and the smallest contributed 7 sites (1.00% of all sites).

**Fig 5:**
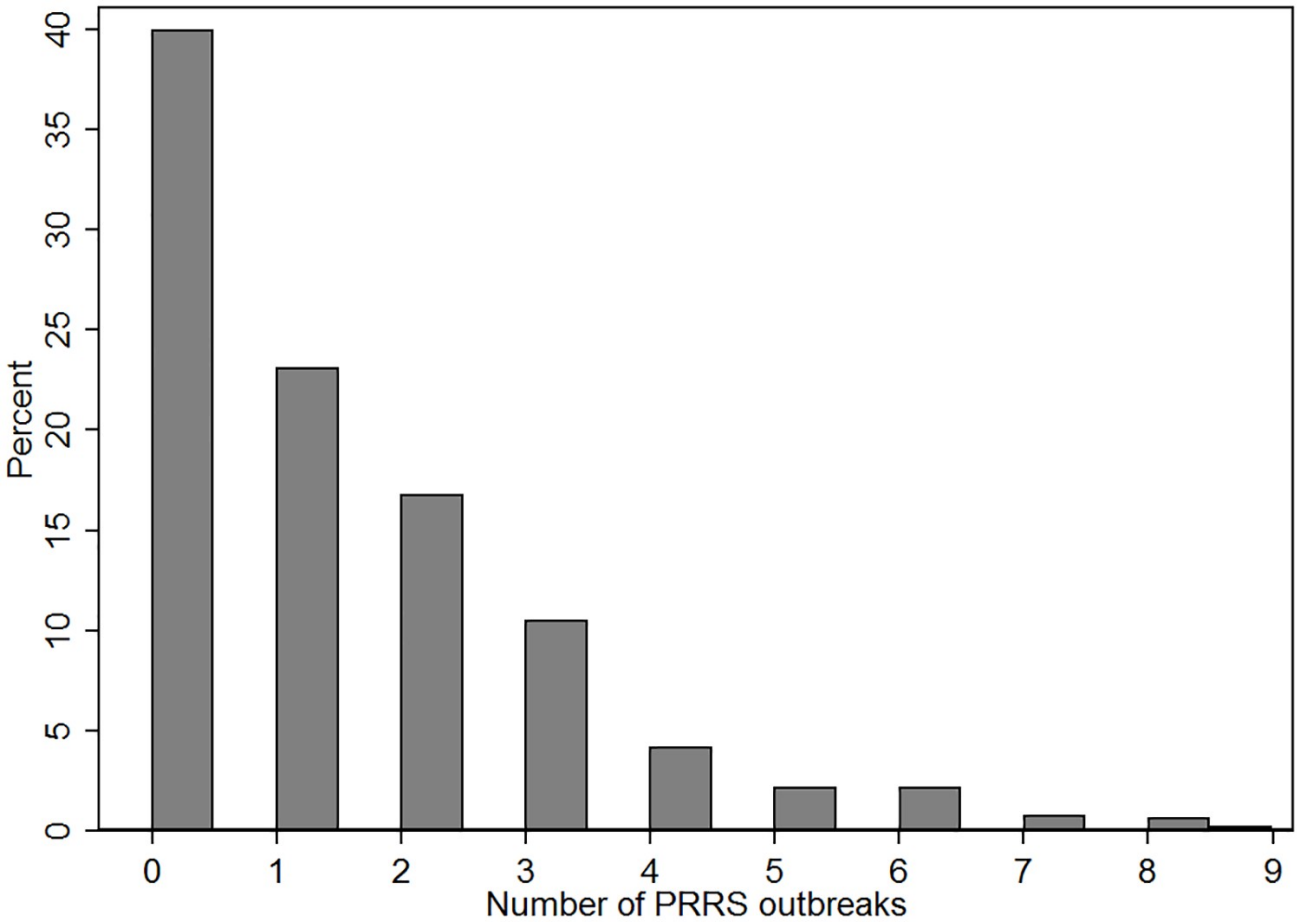
Histogram of distribution of PRRS outbreaks for all swine sites enrolled in the study over the years 2009–2016.

The annual median number of outbreaks per site was 1 (IQR: 2), and data were highly right-skewed (Fig 5). The annual maximum number of outbreaks per farm was 9, and approximately 60.0% of the farms reported at least one outbreak through the study period (n = 424).

Unadjusted risk factor analyses showed significant associations between all predictors of interest and occurrence of PRRS outbreaks, except for land altitude (meters above sea level; Table 1).

Nature and significance of associations were retained in the final multivariable model.

The final model (Table 2) included pig density, number of pigs in the site, land coverage, land slope, and region. There was a positive association between being located in a high dense swine area and herd size, and incidence of PRRS outbreaks (P < 0.01). Being located in a high-slope land (at least approximately 9%) appeared to have a protective effect on the incidence of PRRS outbreaks compared to being located in an area characterized by modest slopes of 2% or less. Land coverage was equally important, where any type of pasture-like cover, needle- or broad-leaved tree cover appeared to be negatively associated with incidence of PRRS when compared to cultivated and managed areas. Interactions were tested in the model and were not significant. The interaction between terrain slope and land cover could not be tested because the model did not converge, likely due to the large number of categories.

**Table 2:** Final multivariable generalized mixed Poisson model; the main outcome modeled herein was the counts of PRRS outbreaks using 706 sow sites as the unit of analysis, and an offset to account for different numbers of years participating in data collection (2009–2016) was used. The model accounted for clustering of swine sites within production systems using a random effect.

Variable	Category	IRR (SE)^1^	95% CI	P-value
Intercept		0.21 (0.05)	(0.13, 0.34)	<0.01
Pig density^2^	Low	Ref		
	High	1.46 (0.11)	(1.23, 1.73)	<0.01
N pigs^3^	Low	Ref		
	High	1.31 (0.11)	(1.11, 1.54)	<0.01
Land coverage	Cultivated, managed	Ref		
	Shrubs, herbaceous cover	0.70 (0.12)	(0.50, 0.98)	0.038
	Trees, needle-leaved/ mixed	0.56 (0.11)	(0.38, 0.82)	<0.01
	Trees, broad-leaved	0.42 (0.14)	(0.22, 0.80)	<0.01
Land slope^4^	<2%	Ref		
	2–4%	1.01 (0.15)	(0.74, 1.36)	0.95
	5–8%	0.77 (0.12)	(0.53, 1.05)	0.10
	9–16%	0.44 (0.10)	(0.28, 0.70)	<0.01
	17–30%	0.18 (0.11)	(0.05, 0.62)	<0.01
Region	Illinois	Ref		
	Minnesota/ Iowa	1.59 (0.23)	(1.20, 2.11)	<0.01
	North Carolina	0.83 (0.29)	(0.42, 1.64)	0.60
	Nebraska	0.70 (0.13)	(0.48, 1.02)	0.06
	Other	0.49 (0.11)	(0.32, 0.75)	<0.01
	Oklahoma	1.28 (0.35)	(0.74, 2.19)	0.38
	Pennsylvania	0.58 (0.34)	(0.18, 1.87)	0.36

^1^Incidence rate ratio (standard error)

^2^Categorized in the median (46 pigs/km^2^)

^3^Categorized in the median (2500 pigs/site)

^4^Land inclination, measured in % or degrees

The random effect variable for production system was significant in the final model (estimate variance: 0.34; SE: 0.133, 95%CI: 0.16, 0.74). Compared to a regular Poisson regression model, the mixed model in which this random effect was incorporated showed a considerable reduction in both the AIC and BIC values, which decreased from 1966.62 and 2039.58 to 1824.49 and 1903.00, respectively. Furthermore, the coefficients for density, number of animals, and a few categories for the slope and region variables changed significantly (>20%), showing the importance of this variable as a confounder.

**Fig 6:**
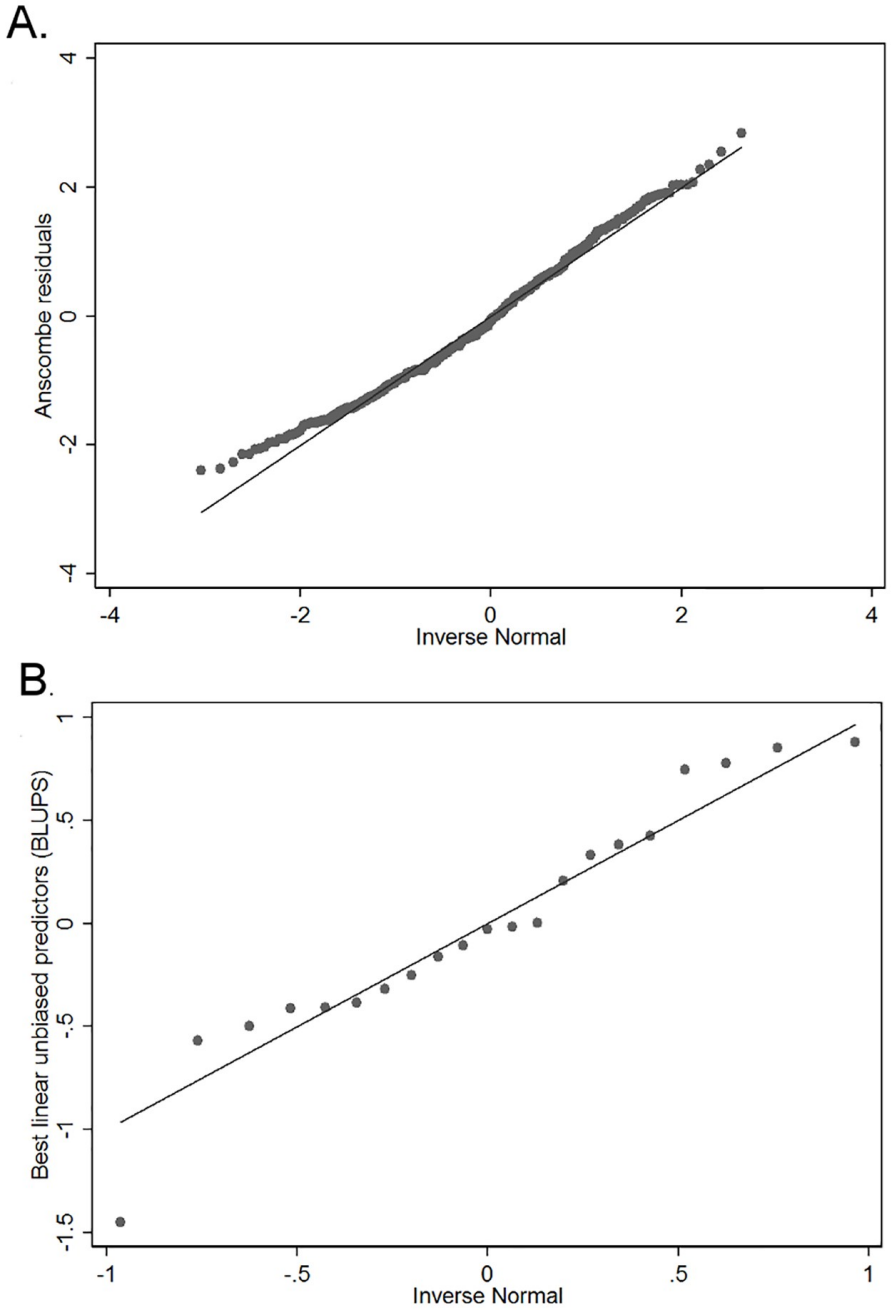
Normal quantile plots for Ascombe residuals (A) and best linear unbiased predictors (BLUPS; B) for the generalized mixed Poisson model used to model the association between demographic and environmental variables of interest and the occurrence of PRRS outbreaks in sow farms across the United States.

Both the Ascombe residuals and BLUPS followed a normal distribution (Fig 6A and 6B). Furthermore, only four observations showed a Pearson residual value higher than 3. Risk factor analysis was repeated using a negative binomial generalized mixed model but the generalized mixed Poisson model presented lower values for both AIC and BIC and was therefore selected.

The Moran’s I test initially detected autocorrelation in the response variable (P < 0.05), which disappeared when pig density was included in the model (P > 0.05), suggesting spatial dependence was related to those factors, and, most important, that the associations detected here were independent from that risk.

## Discussion

Here, association of land-related characteristics and incidence of PRRS outbreaks has been reported for the first time in the peer-reviewed literature. The most important insights of this study were that presence of shrubs, herbaceous land cover, and trees appeared to serve as protective factors for PRRS outbreaks, when the swine farm level was located in areas characterized with a 9% slope or higher. Those associations held true after accounting for known confounders and clustering effects that included production system, region, and pig density. One of the main strengths of this study was that a large number of herds were enrolled over a considerable period of time and these herds were located across many swine-producing areas in the United States.

Some of the associations found in the present study have been previously reported for PRRS, such as pig density and number of pigs in the farm being positively associated with PRRS outbreaks [19–21]. Even though the association between being located in a certain region in the United States and incidence of outbreaks was never reported in the peer-reviewed literature, it is not surprising that the region of Minnesota/ Iowa had higher incidence rate compared to the Illinois region (IRR = 1.59, P < 0.01). The Minnesota/ Iowa region is among the highest swine dense regions in the country and the world, and therefore this region would not only be exposed to higher amounts of emission of airborne viral particles from potentially infected herds, but also have higher rates of traffic and transportation besides other unmeasured PRRSV exposures (e.g. comingling of producers and opportunities for cross-contaminations during manure spread, culling, slaughterhouse and other events).

Our study showed that farms located in terrains with slopes of 5% or higher were “protected” from PRRS outbreaks when compared to sites located with terrains of <2%. There were three categories examined that showed a gradient effect (stronger and more statistically significant association as the slope increased), even though the 5–8% category was not statistically significant. Land altitude as defined by meters above sea level did not remain in the final model, which suggests that the inclination of the terrain as compared to the neighboring regions might be the important component as opposed to altitude on itself. That finding is biologically sound, given that slope may influence PRRSV airborne transmission, acknowledging that airflow, pathogen virulence, and exposure intervals may affect disease likelihood of transmission and infection [22].

Even though there is proof-of-concept that long distance airborne transport of PRRS is possible [23, 6], the authors are not aware of any publication that investigated land inclination in the context of between farm transmissibility of PRRS or any other airborne viruses. In a study conducted in 2009 [23], researchers isolated infectious virus from a swine site located approximately 4.7 km from the alleged source herd. This virus had a ≥ 98.8% ORF5 similarity to the PRRSV present on the source population. Noteworthy, these sampling points were reported to be located in a flat topography area and surrounded by what was described as being primarily of grain fields. Developers of airborne transmission models have reported that local topography may play a role in infection of cattle by foot-and-mouth disease virus (**FMDV**). Models showed that even when exposed to similar amounts of airborne virus, not all farms experience an outbreak [24]. It has been reported that viable FMDV from infected pigs may remain airborne for sufficient time to be carried tens of kilometers downwind [25]. But virus viability and ability to be transferred across farms is a complex process likely depending on conditions such as wind speed and direction, air turbulence, period of the day and air humidity. These factors were not available and could not be included in the models for the study presented here; but they might be important and should be considered in future studies. Furthermore, the authors recognize that the possibility of a regional effect on ‘protection’ of sites from PRRS outbreaks could not be ruled out from our study design, considering the level of resolution of the slope raster was approximately 10km.

As anticipated, production system appeared to be an important clustering level. Besides the fact that swine sites within the same production system tend to be located in similar geographical regions, this importance could also be explained by management-related factors that are commonly standardized within systems (e.g. biosecurity measures, animal handling and movement activities, vaccination or exposure strategies for diseases, etc.) [9]. Furthermore, because PRRS is an unregulated infectious disease in the US, control strategies are commonly a system-level decision.

Here, presence of shrubs and herbaceous cover, needle-leaved and broad-leaved trees decreased the incidence of PRRS by 0.70, 0.56 and 0.42, respectively. These associations remained significant after accounting for other risk factors for PRRS. Potential beneficial effects of land coverage have been previously explored for poultry. Planting trees around poultry farms has been utilized for wind breaks, shade, as a visual appealing screen, and to filter airborne and odor emissions [26]. Those results also suggested that planting multiple rows of trees downwind of exhaust fans may help reduce and disperse farm emissions [26]. Various types of trees were able to play the role of a vegetative buffer by trapping aerial ammonia near poultry fans under different temperature conditions [27]. A follow-up study [28] further showed that different types of trees may effectively trap ammonia, particular matter (dust). Reduction in aerial ammonia depended on where plants were located in regards to distance to fans and type of tree (foliage). Particle deposition processes differed substantially according to particle sizes and interactions with various vegetation elements [29], suggesting that vegetation influences risk for spread.

Noteworthy, landscape attributes examined here have been previously implicated as risk factors for vector borne diseases such as West Nile Fever, malaria and dengue in the context of presence or absence of habitats that provide a supportive environment for vectors or reservoir hosts, as well as on the use of land in the context of opportunities for contact between host and pathogen [30, 31].

A number of biases may have affected the results presented here, including misclassification bias. Certainly, outputs may have been misclassified due to imperfect sensitivity of outbreak detection and reporting, and the value of exploratory variables (e.g. land coverage) may have also changed since the time when the raster was built. However, authors believed that such bias was non-differential and, thus, on average, results were not affected. On a similar consideration, data quality may be an issue for capture of exploratory variables of interest that were extracted from publicly available rasters. Such limitation is difficult to address under the current conditions of this study, and authors aimed to provide references for each raster so that readers are able to refer to those and consider the data source appropriately. More specifically for the land coverage raster, the authors recognize that more recent data is currently available; but the authors would argue that this is a never-ending limitation of this study design, in which assessment of the land was taken at one point in time only. It is important to consider that the current study does not allow for the proof of causality among the variables examined, and that subsequent studies are needed that would incorporate precise measures of land characteristics at time at which outbreaks occur.

Unknown confounders may have also affected the results, such as farm biosecurity-related factors, and animal movements. Those data were not made available to us. However, biosecurity characteristics are relatively standard for North American sow farms, particularly within systems. For those reasons, the random effect imposed at the system level is expected to have accounted, at least in part, for those potential confounders. Finally, the population participating in the SHMP is biased because participants are enrolled on a volunteer basis, which is a type of selection bias. However, population is represented across different U.S. states, in all major swine producing areas (Fig 2A and 2B), and includes a large portion (42%) of the U.S. sow population. For that reason, the assessed population is expected to be reasonably representative of the target population.

An additional important point to consider when generalizing results is that sow herds was the target population of this study, and growing pig populations were not included. It would be interesting to extend this analysis to a larger population including growing pig sites. We recognize that risk factors for PRRS might differ within these populations of sites, and it is generally accepted that breeding sites commonly invest more in prevention and control of outbreaks. Underlying immunity of the swine sites at time of break (and time spent under that status) is another factor that should be taken into account in future studies.

## Conclusions

In conclusion, highly inclined terrains were associated with fewer PRRS outbreaks in US sow farms, as was the presence of shrubs and trees when compared to cultivated/ managed areas. Influence of terrain characteristics on spread of airborne diseases, such as PRRS, may help to predicting disease risk, and effective planning of measures intended to mitigate and prevent risk of infection.
